# Relationships between Emotion, Acceptance, Food Choice, and Consumption: Some New Perspectives

**DOI:** 10.3390/foods9111573

**Published:** 2020-10-29

**Authors:** Witoon Prinyawiwatkul

**Affiliations:** School of Nutrition and Food Sciences, Louisiana State University Agricultural Center, Baton Rouge, LA 70803, USA; wprinya@lsu.edu

**Keywords:** food-evoked emotions, sensory liking, consumer acceptance, purchase intent, food choice, food intake and consumption

## Abstract

Food is more than just a source of nutrients—it also provides basic pleasure as well as aesthetic experiences. A number of studies have reported that acceptance, food choice, and consumption are affected by a large number of factors, including both intrinsic and extrinsic factors and cues, as well as consumer characteristics. Food-elicited emotions are becoming a critical component in designing products that meet consumers’ needs and expectations. Several studies have reported emotional responses to food and their relationships to product acceptability, preference, and choice. This Special Issue brings together a small range of studies with a diversity of approaches that provide good examples of the complex and multidisciplinary nature of the subject matter.

## Introduction

Modern consumers are becoming ever-more health conscious and more educated about what constitutes their foods. Many consumers want healthier and safer versions of retailed food products. These consumers are concerned about the health benefits or risks associated with food consumption. Globalization has enabled consumers to be exposed to various cuisines which can be readily available to them. With the world population increasing rapidly, alternative food sources and mass food production will be needed to support sustainability and safety. The questions are: “Would consumers be willing to consume this? How do they feel about the food they eat? Do they like and will they purchase it?” Based on many studies, acceptance, food choice, and consumption are affected by a large number of factors, including both intrinsic and extrinsic factors and cues, as well as consumer characteristics.

It is known that food elicits emotion. Measuring food-evoked emotions is topical in sensory and consumer sciences. Emotions are becoming a critical component in designing products that meet consumers’ needs and expectations. Emotional profiles may effectively differentiate products with similar sensory characteristics and hedonic ratings, hence, they may provide additional information that goes beyond traditional hedonic ratings, and provide more insight toward food choice. Several studies have reported emotional responses to food, and their relationship to product acceptability and purchase intent. Appropriate health benefit information has also been reported to impact emotion, purchase decisions and food choices. Human senses and cues play an instrumental role in food choice and intake, emotion and product acceptance, hence, understanding their roles and importance is critical.

This Special Issue of Foods aimed to present both original and cutting-edge research contributing to a deeper understanding of relationships between food-evoked emotion, food choice, acceptance, and consumption.

Consumers use various intrinsic and extrinsic informational cues to form impressions about the quality of food products and to make subsequent purchase and consumption decisions. One study utilized a multifaceted approach to develop an emotional and wellness profile associated with ready-to-eat (RTE) salads [1]. Several emotional terms were proven to distinguish among RTE salad samples, depending on the prominent visual cues presented to consumers. The effects of both intrinsic and extrinsic visual cues on emotions and the liking of RTE salads were evident. The authors concluded that liking intrinsic visual characteristics of salads may moderate the effects of extrinsic cues. Providing additional product information, such as naming or information on packaging, may help reinforce positive tendencies towards making healthy food choices and purchasing intentions.

Maize tortilla is known to be a staple food in Mexico. Contemporary commercial-scale production of tortillas makes use of instant maize flours and specialized machines, which leads to drastic changes in sensory characteristics. One study was performed to investigate consumer preference and choices related to tortillas; comparing artisanal hand-made ones vs. those produced mechanically [2]. The authors reported that the sensory profile of the artisanal ones was better and more nutritious compared to the others. Differences between women’s and men’s preferences and purchase decisions were observed; men considered taste, while women considered the maize type as a critical factor. Consumers’ choices for tortilla are important for producers, so the results of this study may help tortilla producers to better understand some quality characteristics of their products that affected consumer preferences. Alternate research has investigated the hamburger. One study connected the rationale (food values) and positive anticipated emotions to different attitudes in order to predict purchase intent of hamburgers [3]. The authors attempted to identify which emotions, food values and types of attitudes significantly and positively influenced purchase intent. They concluded that the positive anticipated emotion (contentment, excited and satisfied) positively influenced attitudes toward the brand, attitude toward eating, and intention to buy a hamburger at a fast-food restaurant.

There are two studies in this Special Issue that are related to alcohol and wine consumption. Only a few studies have been devoted to address the impact of both intrinsic (taste, aroma, flavor, etc.) and extrinsic (brand, labelling, price, etc.) factors in conjunction with consumer characteristics and attitudes on preference, choice and consumption of wine. One study was performed to analyze preferences of sherry wines as influenced by gender, knowledge, and interest in wine, particularly among young consumers between 18 and 30 years old [4]. The authors concluded that there was a relationship between prior knowledge of and interest in wine culture and wine consumption in young adults. The findings would allow wine producers to have a better idea of young consumer perceptions towards wine, and how to promote a non-abusive consumption of wine among young adults. Contrarily, another study was devoted to alcohol consumption in young people, which is a public health problem [5]. From a psychological point of view, personality variables are clearly associated with alcohol consumption. The authors stated that no data have been found regarding the relationship between emotional intelligence (EI, the ability to perceive, evaluate and express emotions accurately), impulsivity, and alcohol consumption. They concluded that young people with a low level of EI tended to be more impulsive and had poor handling of their emotions, leading to a possible increased risk of alcohol consumption. The findings demonstrated some variables that could prevent alcohol consumption in young people.

In another study, the relationships among ethnic food consumption, food neophobia, consumers’ openness to different cultures, and consumer sociodemographic characteristics were evaluated using an online survey with Italian consumers [6]. The authors concluded that consumers with food neophobia would not consume ethnic food, while those with openness to different cultures would. Some sociodemographic variables associated with food neophobia included gender, age, education, marital status (with or without children), and income. The findings from this study may be useful in promoting a diversity of healthy ethnic diets.

The last study utilized massive online textual data to evaluate public opinions on the safety of take-out foods in China between 2015–2018 [7]. The collected data were mined and analyzed using a dictionary-based emotional analysis of text, followed by emotional time series analysis to reveal emotional trends and tendencies. The authors concluded that during this four-years period, the trends of topics/discussions, which carried positive and negative emotions, on take-away food safety were similar, although the number of positive posts about food safety were much higher than the negative posts. The findings would offer insights for government and industry stakeholders as to how to promote safety of take-away food.

The editor hopes that the readers will find this Special Issue insightful, interesting and useful for future research. The diversity of both the content and the methodologies presented in this Special Issue should inspire and encourage future exploration of multidisciplinary research collaboration, which would lead to a better understanding of the complex relationships between emotion, acceptance, choice, and consumption of food.
